# TREM-1^low^ is a novel characteristic for tumor-associated macrophages in lung cancer

**DOI:** 10.18632/oncotarget.9639

**Published:** 2016-05-26

**Authors:** Guangbo Zhang, Hongmei Liu, Jian Huang, Siwen Chen, Xudong Pan, Haitao Huang, Ling Wang

**Affiliations:** ^1^ Clinical Immunology Laboratory, The First Affiliated Hospital of Soochow University, Suzhou, 215007, China; ^2^ Department of Special Procurement Ward, The First Affiliated Hospital of Soochow University, Suzhou, 215007, China; ^3^ Department of Emergency, The First Affiliated Hospital of Soochow University, Suzhou, 215007, China; ^4^ Department of Thoracic Surgery, The First Affiliated Hospital of Soochow University, Suzhou, 215007, China

**Keywords:** TREM-1, tumor-associated macrophages, tumor microenvironment, lung cancer

## Abstract

**Objective:**

To explore the expression feature and biological functions of TREM-1 on tumor-associated macrophages (TAMs) in lung cancer.

**Results:**

The levels of TREM-1 on tissue-infiltrating monocytes/macrophage from tumor nest were significantly lower than those from nonturmor tissue or peripheral blood samples. Clinical analysis indicated that the levels of TREM-1-related TAMs were significantly decreased during cancer stages progression. The tumor-bearing mouse model further confirmed that the expression of TREM-1 on TAMs was significantly decreased with tumor growth. In addition, we found the activation of TREM-1 could significantly enhance the secretion of IL-1β by TAM *in vitro*. Furthermore, T-bet but not Eomes was found to be the key transcription factor for the TREM-1 expression on monocytes/macrophage.

**Methods:**

A total of 40 patients with non-small cell lung cancer (NSCLC) were enrolled in this study. The expression characteristics of TREM-1 in blood and tissue-infiltrating monocytes/macrophage were examined by flow cytometry analysis. After the treatment of TREM-1 antibody, which is an agonist of TREM-1, cytokines secreted by TAM were then analyzed. In LLC-tumor bearing mouse model, we further investigated the dynamic expression feature of TREM-1 on macrophage with tumor growth. Moreover, we explored the transcription factor for regulating TREM-1 expression on monocyes/macrophage with wildtype, T-bet Ko or Eomes Ko mice.

**Conclusion:**

The levels of TREM-1 were remarkably decreased during tumor progression. The low expression level of TREM-1 might be a characteristic for TAMs in lung cancer.

## INTRODUCTION

TAMs are major component of leukocytic infiltrate of tumors and play a pivotal role for tumorigenesis, invasion and metastasis in various solid tumors [1–2]. According to its phenotypes and functions, monocyte/macrophage could be divided into two opposing subgroups: M1 and M2 types [2]. Tumor microenvironment was determined through the mutual conversion between M1 or M2 macrophages. Generally, M1 type macrophages refer to the “classically-activated” macrophage that emerge during cell-mediated immune responses and play anti-tumor roles, whereas M2 type macrophages are the majority of TAMs, which have anti-inflammatory and pro-tumoral properties [3–5]. Their dynamic balance is essential for tumor development [4–5]. Depending on the tumor entity and the prevalent polarization status, macrophages can be associated with a favorable or unfavorable clinical outcome [6]. Therefore, identification for the phenotype of monocytes/macrophages in tumor condition would be important to understand their roles in tumor progression.

As a transmembrane receptor complex, triggering receptor expressed on myeloid cells-1 (TREM-1) is a protein, which consist of a single extracellular immunoglobulin-like domain, a transmembrane region, and a short cytoplasmic tail [7, 8]. After TREM-1 cross-linking, the phosphorylated DNAX activation protein 12 (DAP12) can recruit and phosphorylate growth receptor binding protein 2 (GRBP-2) and phosphatidylinositol-3 kinase (PI3K) to amplify Toll-like receptors (TLRs) [9]. In presence of LPS, TREM-1 can enhance neutrophils or monocytes/macrophages to secret myeloperoxidase, monocyte chemoattractant protein-3 (MCP-3), IL-8, macrophage inflammatory protein-1α (MIP-1α), monocyte chemoattractant protein-1 (MCP-1), and granulocyte-monocyte colony–stimulating factor (GM-CSF) [7, 10]. Thus, TREM-1 would be essential for amplifying inflammatory responses in bacterial infections, fungal infections, or sepsis [7, 8, 11]. Amplification of inflammation is the best-characterized function attributed to TREM-1[12]. Inhibition of TREM-1 could prevent hyper-responsiveness and death during various experimental septic shock models, suggesting that TREM-1 works as an amplifier of immune responses [13, 14, 15].

Based on the previous data, TREM-1 should to be an anti-tumor molecule through enhancing immune responses. However, recent studies indicated that TREM-1 could promote tumor progression. TREM-1 is expressed by Kupffer cells (KC) and regulates the inflammatory response to induce aseptic inflammation in damaged liver tissues, which lead to carcinogenesis subsequently [16]. Ho *et al.* demonstrated that high expression level of TREM-1 on TAMs of the patients with NSCLC was associated with cancer recurrence and poor prognosis [17]. In addition, tumor cells can promote the up-regulation of TREM-1 expression on TAM in tumor environment [17, 18], suggesting that TREM-1 could boost carcinogenesis and cancer progression with an unknown mechanism. Notably, chronic inflammatory state could contribute to tumor progression through inducing tumor angiogenesis [19]. Therefore, all these evidences indicated that the expression and functions of TREM-1 might be different between pathogen infection status and tumor-bearing status.

In this study, we examined the expression of TREM-1 on blood monocytes, tumor and corresponding nontumor tissue-filtrating macrophage in patients with NSCLC. We found that the expression levels of TREM-1 on monocytes/macrophages in tumor microenvironment are significantly lower than those in periphery. Additionally, in NSCLC patients and tumor-bearing mouse model, our results demonstrated that the expression levels of TREM-1 on monocytes/macrophages were significantly decreased during tumor progression. We also found that TREM-1 activation could promote TAM significantly to secrete IL-1β in presence of LPS. Therefore, our findings suggested that the inherent function of TREM-1 might still work as an amplifier of immune responses in tumor microenvironment, but its effects will be gradually receded with the decrease of TREM-1 levels on TAM with tumor progression.

## RESULTS

### TAM shows a TREM-1^low^ phenotype in lung tumor microenvironment

**Figure 1:**
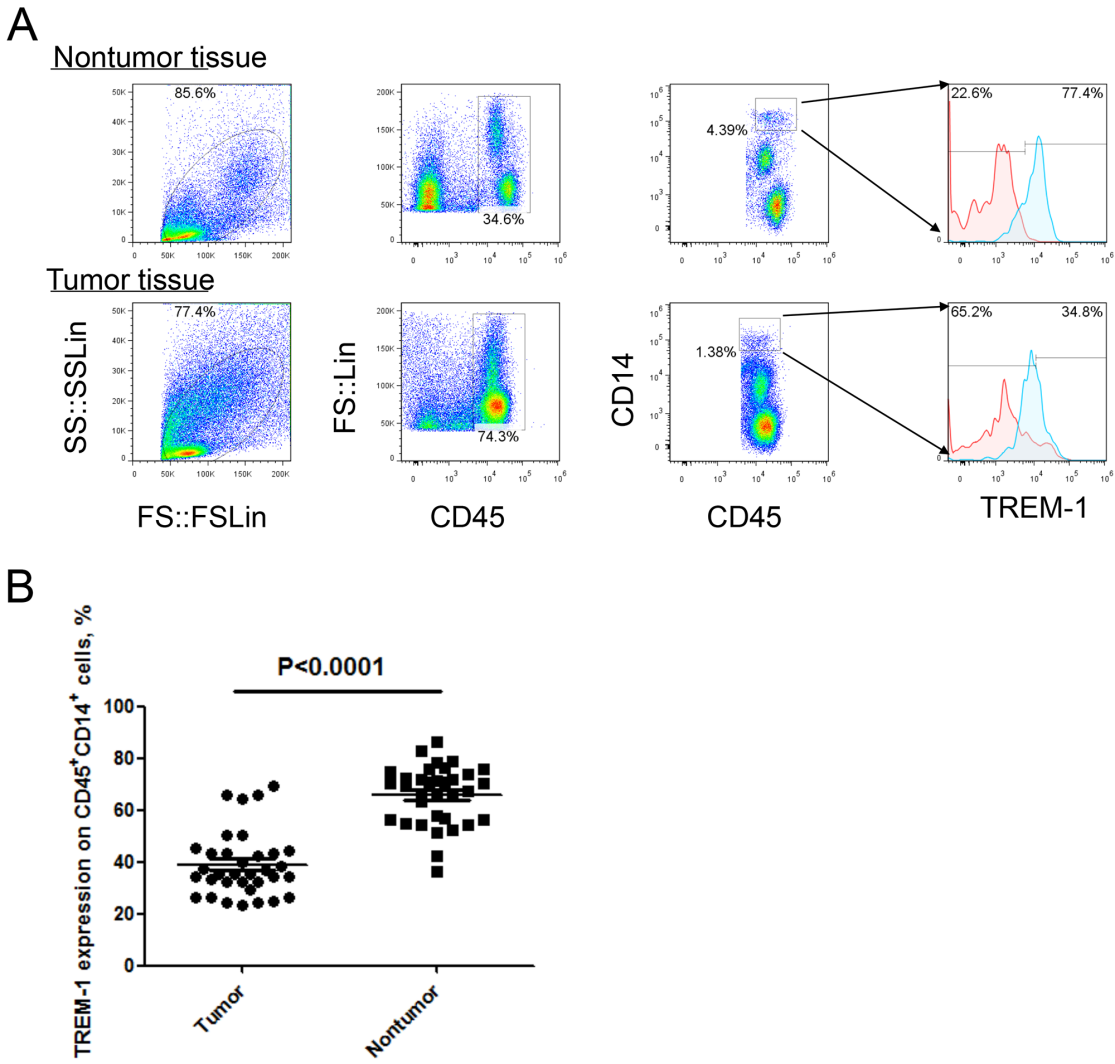
Level of TREM-1 on tumor tissue-infiltrating monocytes/macrophages from patients with NSCLC **A.** Representative dot plots and **B.** summarized data showed the levels of TREM-1 on monocytes/macrophages from the matched tumor and nontumor tissues from NSCLC patients (n=40). Red lines represent IgG control and blue lines represent anti-TREM-1 antibody. Student's *t*-test (paired test) was performed and data are presented as means±SEM.

**Figure 2:**
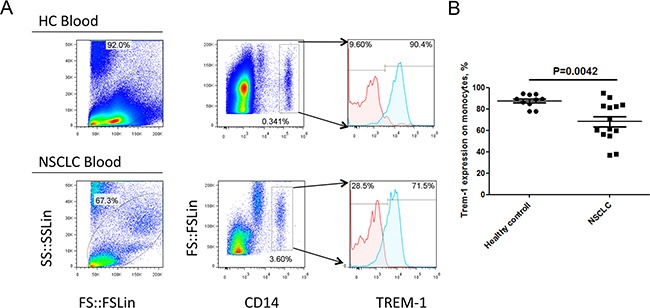
Levels of TREM-1 on blood monocytes from patients with NSCLC and healthy control **A.** Representative dot plots and **B.** summarized data showed the levels of TREM-1 in blood CD14^+^ leukocytes from healthy donors (n=10) or NSCLC patients (n=14). Red lines represent IgG control and blue lines represent anti-TREM-1 antibody. Student's *t*-test (unpaired test) was performed and data are presented as means±SEM.

To investigate the expression feature of TREM-1 on TAM in tumor microenvironment, we detected the levels of TREM in tumor tissues and distal normal lung tissues with flow cytometry. Our results demonstrated that the level of TREM-1 on CD45^+^CD14^+^ monocyte/macrophage from tumor tissue displays a significantly lower than that from corresponding distal nontumor lung tissues (Figure 1). Besides, we found the level of TREM-1 on periphery circulating monocytes is also lower in patients with NSCLC than that in physical examination counterparts (Figure 2). Notably, further analysis indicated that TREM-1 on tumor tissue-derived monocytes/macrophage was significantly lower compared with that on peripheral blood monocytes from patients with NSCLC (Supplementary Figure S1). Therefore, our data indicated that TREM-1^low^ may be a novel characteristic for TAM in human lung cancer.

### The levels of TREM-1 on TAM were decreased with tumor progression

**Figure 3:**
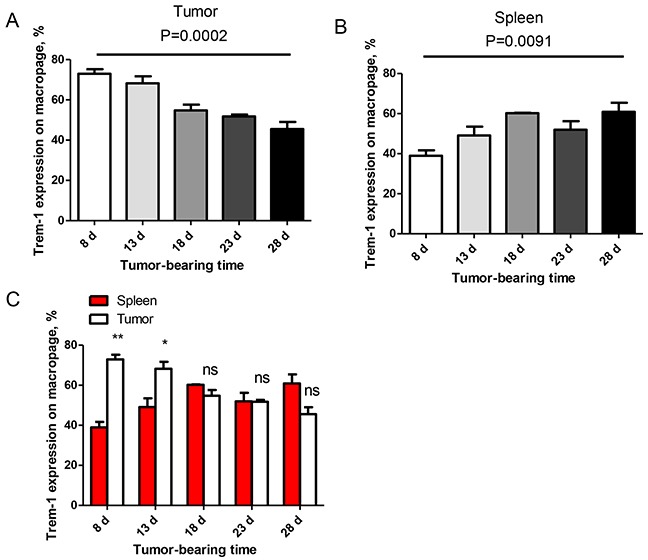
Levels of TREM-1 on monocytes/macrophages were decreased during progression of tumor in a mouse lung carcinoma model **A, B.** TREM-1 levels on tumor and spleen-infiltrating CD11b^+^F4/80^+^ monocytes/macrophages were detected in LLC-bearing mice. One-way ANOVA was performed and data are presented as means±SEM (n=3/one time point). **C.** In early stage, tumor microenvironment inhibit TREM-1 expression on CD11b^+^F4/80^+^ monocytes/macrophages *in vivo*. One-way ANOVA was performed and data are presented as means±SEM (n=3/one time point). * represents *P*<0.05, ** represents *P*<0.01, ns represents no significance.

As shown in Table 1, the TREM-1 levels on monocytes/macrophages gradually reduced with the advance of tumor stage and lymph node metastasis, suggesting that TREM-1^low^ on TAM might be a novel characteristic for advanced stage of lung cancer. We next explored the clinical significance of the levels of TREM-1 on TAM. We therefore generated a tumor-bearing mouse model with cell line LLC to confirm this hypothesis. The dynamic expression of TREM-1 was detected on CD11b^+^F4/80^+^ macrophage isolated from spleen and tumor tissues by flow cytometer. We found that the levels of TREM-1 on tumor tissue-derived macrophage gradually decreased with tumor growth (Supplementary Figure S2, Figure 3A). whereas the TREM-1 levels on macrophage from spleen exhibited an alternation with opposite direction and significantly increased with tumor growth (Supplementary Figure S2, Figure 3A and 3B). Comparative analysis indicated that TREM-1 levels on macrophage from tumor tissue samples were significantly higher than those from spleen samples in early stage (at the 8^th^ and the 13^th^ day after tumor-bearing) of tumor progression (Figure 3C). However, along with tumor growth, the difference gradually disappears from 18^th^ day after of tumor-bearing (Figure 3C). All these evidences indicated that the effects of tumor-bearing on TREM-1 expression might be strikingly different between on periphery circulating monocyte/macrophage and tumor-tissue infiltrating monocyte/macrophage.

**Table 1:** TREM-1 on TAM is associated with lung cancer progression

Character	n	TREM-1 expression		P
		high	low	
**Sex**				
Male	25	13	12	0.744
Female	15	7	8	
**Age**				
≤60	14	9	5	0.1848
>60	26	11	15	
**Histological subtype**				
Squamous cell carcinoma	16	8	8	0.8813
Adenocarcinoma	19	10	9	
others	5	2	3	
**Tumor size**				
≤ 3 cm	16	7	9	0.7474
> 3cm	24	13	11	
**Lymph node metastasis**				
0	21	15	6	0.0048
1+2	19	5	16	
**Tumor stage**				
I+II	23	16	7	0.0095
III	17	4	13	
**Smoking**				
yes	23	14	9	0.2003
no	17	6	11	

### TREM-1 activation can enhance TAM to secrete IL-1β in tumor microenvironment

**Figure 4:**
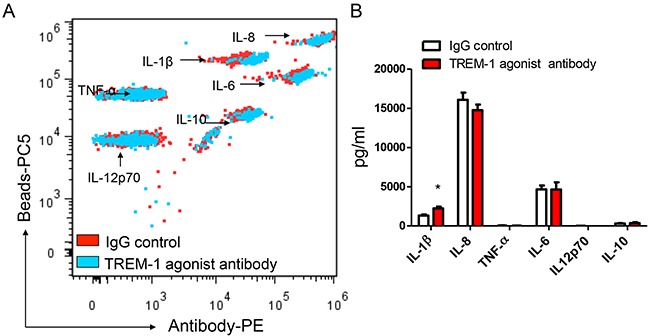
TREM-1 activation promotes TAMs to amplify inflammatory cytokine IL-1β in tumor microenvironment The supernatants of TAMs stimulated with anti-TREM-1 agonist monoclonal antibody or IgG isotype control were determined for the levels of IL-1β, IL-6, IL-8, IL-10, IL-12p70, and TNF-α through CBA by flow cytometry analysis **A.** and summarized data were shown (n=4); **B.** Student's *t*-test (paired test) was performed and data are presented as means±SEM (n=4).

**Figure 5:**
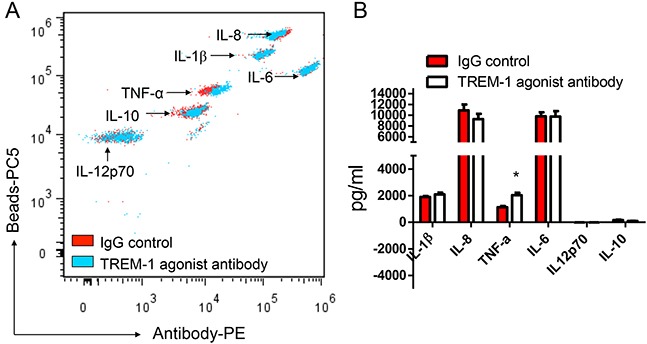
TREM-1 activation promotes blood monocytes to amplify inflammatory cytokine TNF-α The supernatants of blood monocytes stimulated with anti-TREM-1 agonist monoclonal antibody or IgG isotype control were determined for the levels of IL-1β, IL-6, IL-8, IL-10, IL-12p70, and TNF-α through CBA by flow cytometry analysis **A.** and summarized data were shown **B.** Student's *t*-test (paired test) was performed and data are presented as means±SEM (n=3).

To reveal the biological functions of TREM-1 in tumor microenvironment, we then sorted TAM from lung cancer tissues by flow cytometry. In presence of LPS, the purified TAM was stimulated with anti-TREM-1 agonist monoclonal antibody or IgG isotype control for 24 hrs. Our results indicated that the activation of TREM-1 could significantly enhance IL-1β secretion in TAM (Figure 4). However, the level of other cytokines including IL-6, IL-8, IL-10, IL-12p70 or TNF-α, displays no statistically difference between TREM-1 activated and IgG control group. In addition, we obtained different result using blood monocytes isolated from healthy people. With TREM-1 aviation, blood monocytes could significantly increase TNF-α secretion but not IL-1β (Figure 5).

### T-bet can increase TREM-1 expression on monocyte/macrophage

**Figure 6:**
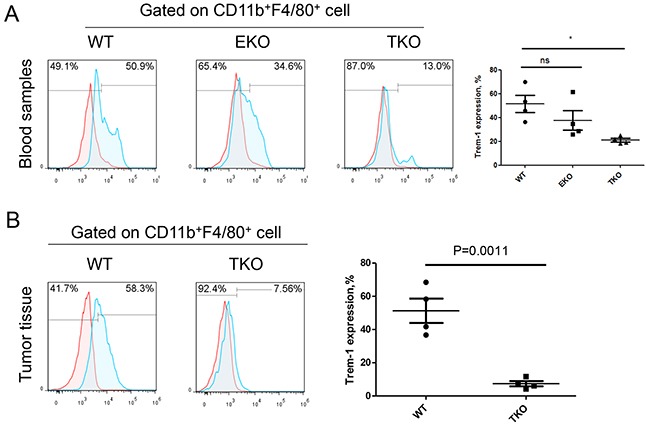
T-bet is involved in the regulation of TREM-1 expression on monocytes/macrophages Representative dot plots (right) and summarized data (left) of TREM-1 expression on **A.** blood or tumor-tissue infiltrating **B.** CD11b^+^F4/80^+^ monocytes/macrophages of WT, T-bet ^−/−^ and Eomes^−/−^ mice were shown. Red lines represent IgG control and blue lines represent anti-TREM-1 antibody. The data were expressed as mean±SEM. The statistical method used for this analysis was one way ANOVA. ^*^ represents *P*<0.05, ns represents no significance.

T-bet and Eomes are key transcription factor in the regulation of T cell and monocytes subset differentiation [19], but their effects on the regulation of the TREM-1 expression still remain unclear. We then analyzed the expression level of TREM-1 on monocytes/macrophage isolated from WT mice, TKO mice, or EKO mice, respectively. In peripheral blood, we found that CD11b^+^F4/80^+^ monocytes/macrophage derived from TKO but not EKO mice have the significantly lower level of TREM-1 than those from WT mice (Figure 6A). Also, the effect of T-bet on TREM-1 expression was further confirmed in tumor microenvironment (Figure 6B), indicating that T-bet might be essential for the increase the TREM-1 expression on monocyte/macrophage.

## DISCUSSION

Previous studies indicated TREM-1 was constitutively expressed by monocytes in human peripheral blood, and increased strikingly by LPS stimulation [7]. As we known, TREM-1 could amplify the inflammatory response and inhibition of TREM-1 could decrease death in various experimental septic shock models [7–12, 14, 15]. As an amplifier of immune responses, TREM-1 should be considered as an anti-tumor molecule during tumorigenesis and tumor development [23]. However, recent studies demonstrated that TREM-1 might be involved in tumor progress. High expression levels of TREM-1 were reported to be associated with tumor recurrence and poor prognosis of patients with NSCLC [17]. Wu *et al.* showed that TREM-1 expressed by KC is a crucial factor in the development and progression of liver cancer [16]. In addition, Inhibition of TREM-1 by short hairpin RNA (shRNA) in macrophages could suppress cancer cell invasion *in vitro* [18]. As previously described, TREM-1 is known as a key molecule for the triggering and amplification of inflammatory response to stimulate proinflammatory cytokines secretion [7, 8]. Different from chronic inflammation, excessive inflammatory response should play an anti-tumor role [21–23]. Thus, we want to reveal the expression and functions of TAM-related TREM-1 in tumor microenvironment.

In this study, we found that macrophages isolated from cancer tissue comparing with from blood show a character of IL-12p70^low^IL-10^high^ (Figure 4 and Figure 5). Besides, biological functional analysis indicated TAMs significantly increased proinflammatory cytokine IL-1β through the activation of TREM-1 (Figure 4), suggesting that TREM-1 on TAMs still play an amplifier of inflammation. In clinical sample analysis, we found that the level of TREM-1 on tumor tissue-macrophage is statistically lower than that on corresponding adjacent lung tissue-macrophage or blood monocytes (Figure 1 and Figure 2). Notably, further analysis indicated that tumor tissue-derived macrophage from patient in early stage showed a significantly higher level than that in advanced stage during tumor progression, while the similar results were also obtained in tumor-bearing mouse model (Table 1 and Figure 3). Moreover, along with tumor growth, the levels of TREM-1 gradually decreased on macrophage of tumor tissues (Figure 3A and 3B). Interestingly, our results demonstrated that TREM-1 levels gradually increased on spleen macrophage with tumor growth, which is an opposite trend in periphery during tumor progression (Figure 3C). We think the different state of immune between periphery (spleen) and tumor microenvironment (tumor tissue) may lead to the difference of TREM-1 expression during tumor progression. All these results indicated that TREM-1 expression showed a dynamic alternation on macrophage during tumor progression, suggesting that the expression levels of TREM-1 might be a novel characteristic for evaluating macrophage state in tumor microenvironment.

Since our results indicated that the decreasing TREM-1 expression on TAMs in tumor tissue is associated with cancer metastases and tumor progression in the advanced stage, we expected that the lower levels of TREM-1 on TAMs might be a promising prognostic biomarker. However, Ho *et al.* previously showed that the high expression levels of TREM-1 in tumor tissue should be an independent factor and represent a poor prognosis [13]. We thought a “decreased” level of TREM-1 may offer a more suitable “chronic inflammation-related soil” for tumor growth in tumor microenvironment. In contrast, TREM-1^high^ monocyte/macrophage might ignite anti-tumor responses in periphery.

In summary, we found the different pattern of TREM-1 expression from previous reports in patients with NSCLC and tumor-bearing mouse model. We found the decreasing level of TREM-1 on TAM with tumor advance by flow cytometry analysis. Therefore, we expected that TREM-1^low^ might be a novel characteristic for TAMs in lung cancer, and its intervention might provide a macrophage-centered clinical therapeutic strategy for cancer treatment.

## MATERIALS AND METHODS

### Patients and tissue samples preparing

Fresh tumor tissue and the corresponding nontumor tissue samples were obtained from 40 untreated patients with pathologically confirmed NSCLC at the Department of Thoracic Surgery, the First Affiliated Hospital of Soochow University from Oct. 2012 to Jan. 2014. Lobectomy with radical mediastinal and hilar lymphadenectomy was performed on all patients. The evaluation of tumor stages was based on the tumor, node, and metastasis classifications (TNM) of the International Union against Cancer. Blood samples were collected by venipuncture technique from patients before operation and from healthy donors who underwent physical examination as control. Tumor tissues were taken from areas of solid tumor tissues lacking the gross aspect of massive necrosis. The corresponding nontumor tissue samples were taken at least 5 cm away from the visible tumor margin. Fresh tissue samples were used for the isolation of tissue-infiltrating leukocytes. The clinical characteristics of all patients are summarized in Table 1. This study was approved by the ethics committee of the First Affiliated Hospital of Soochow University (Suzhou, Jiangsu Province, China) for clinical investigation, and written informed consent was obtained from patients or their relatives before enrollment.

### Preparation of single cell suspensions

Single cell suspensions of tumor or nontumor sample were obtained after digestion [20]. Briefly, tissues were cut in pieces and placed in petri dishes containing 0.1% Collagenase IV (Invitrogen, Thermo Fisher Scientific Inc., Grand Island, NY, USA) and incubated at 37°C for 60 min. Samples were then grinded, and passed through a 150-μm mesh and a 30-μm filter (Miltenyi Biotec; Bergisch Gladbach, Germany) for Fluorescent-activated cell sorting (FACS) or directly analyzed by flow cytometry.

### Mice treatment

6–8-week-old male C57BL/6 mice were purchased from Changzhou Cawens Laboratory Company (Changzhou, Jiangsu, China). The Eomes gene knocked out (Eomes ^−/−^, EKO) mice and T-bet gene knocked out (T-bet ^−/−^, TKO) mice were the generous gifts kindly provided by Professor Bin-Feng Lu (University of Pittsburgh, Pittsburgh, PA, USA). All the experimental animals were raised under specific pathogen-free conditions in the animal facility at the Laboratory of Clinical Immunology of The First Affiliated Hospital of Soochow University (Suzhou, Jiangsu Province, China). All animal work was performed using an institutional protocol that was approved by the Animal Care and Use Committee of Soochow University.

### Tumor-bearing mouse model

For tumor model experiments, mice were challenged with 2×10^6^ LLC i.d. and tumor samples were removed for analysis around day 8, 13, 18, 23, 28 mimicking tumor progression, respectively. Moreover, we obtained peripheral blood or tumor tissues from healthy mice, TKO mice, or EKO mice, respectively. We also analyzed the expression levels of TREM-1 after labeled with CD11b^+^F4/80^+^ cells isolated from the indicated mice by flow cytometric analysis.

### Antibodies and flow cytometric analysis

Blood leukocytes and tissue-infiltrating leukocytes were stained with fluorochrome-conjugated monoclonal antibodies and then analyzed by multicolor flow cytometry. Isotype-matched antibodies were used with all the samples as controls. The antibodies used in this study are listed as follows: anti-human-CD14-FITC (#301804), anti-human-CD45-PE/Cy7 (#304016), anti-mouse-CD11b-FITC (#101206), and anti-mouse-F4/80-PE/Cy7 (#123114) were ordered from BioLegend (San Diego, CA, USA). Anti-human-TREM-1-PE (#FAB1278P) and anti-mouse-TREM-1-PE (#FAB1187P) were purchased from R&D Systems (Minneapolis, MN, USA). All of the samples were detected on a FC-500 machine (Beckman Coulter; Fullerton, CA, USA) and analyzed using FlowJo software (Stanford University, Stanford, CA, USA).

### Cytokine analysis

CD45^+^CD14^+^TAM were purified from tumor tissue-infiltrating leukocytes and CD14^+^ monocytes were purified from healthy control by using the Beckman Coulter MoFlow^TM^ cell sorting system (Beckman Coulter; Fullerton, CA, USA) for the following experiments. Purified TAM or monocytes (1×10^5^/ml) were cultured with RPMI 1640 medium (Invitrogen, Thermo Fisher Scientific Inc.; Grand Island, NY, USA) and treated with mouse anti-human TREM-1 agonist monoclonal antibody (4 μg/ml, MAB1278; R&D Systems; Minneapolis, MN, USA) or with mouse IgG1 protein (4 μg/ml, MAB002; R&D Systems; Minneapolis, MN, USA) in presence of LPS (1 μg/ml, Sigma-Aldrich; St. Louis, MO, USA) for 24 hrs. After the treatment of stimulation, the supernatants were collected for analysis of cytokines such as IL-1β, IL-6, IL-8, IL-10, IL-12p70 or TNF-α with a human inflammation Cytometric Bead Array (CBA) kit (#551811, BD Biosciences; Franklin Lakes, NJ, USA), respectively. Tests were performed according to the manufacturer's instructions available online. The six bead populations are resolved in a red channel of FC-500 flow cytometer. For each set of experiments, a standard curve was generated. The results were expressed as pg/ml and then analyzed for their relative expression (control versus treated samples). The lower limit for detection for each cytokine was determined as 10 pg/ml.

### Statistical analysis

Statistical analysis for normally distributed values was performed using Student's *t* test or ANOVA. Non-normally distributed values, as assessed by the Kolmogorov-Smirnov test, were analyzed by the Mann-Whitney *U* test. Fisher's exact test was performed for clinical significance analysis. Statistical analyses were performed using GraphPad Prism 5.0 software package (GraphPad Software, Inc.; San Diego, USA). The values of *P*<0.05 were considered to be statistically significant.

## SUPPLEMENTARY FIGURES


