# Supplementary material for: TREM-1low is a novel characteristic for tumor-associated macrophages in lung cancer
Source: Oncotarget. 2016 May 26;7(26):40508–17. doi: 10.18632/oncotarget.9639 (PMC5130024; doi:10.18632/oncotarget.9639)
Supplement: Supplementary file 1 [file oncotarget-07-40508-s001.pdf]

TREM-1^{low} is a novel characteristic for tumor-associated macrophages in lung cancer

SUPPLEMENTARY FIGURES

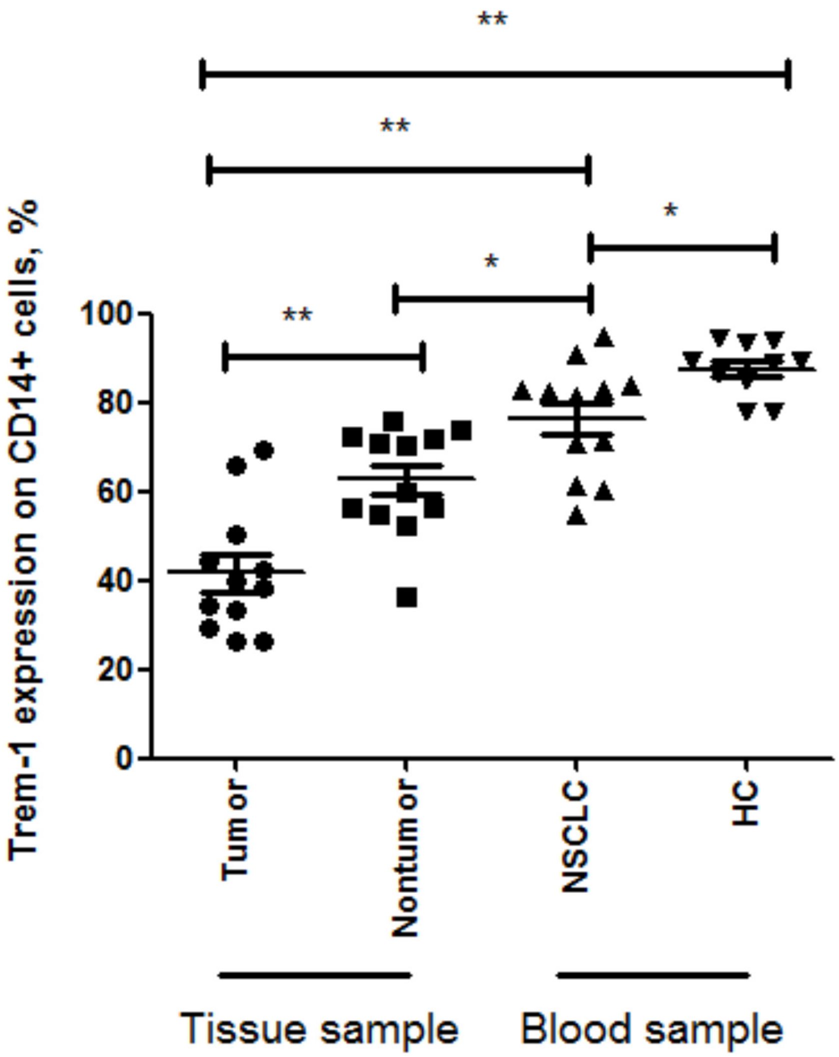

Supplementary Figure S1: Analysis of TREM-1 on blood or tissue-infiltrating monocytes/macrophages. Representative dot plots and summarized data. Mann Whitney test was performed and data are presented as mean ±SEM. * represents $p<0.05$, ** represents $p<0.01$.

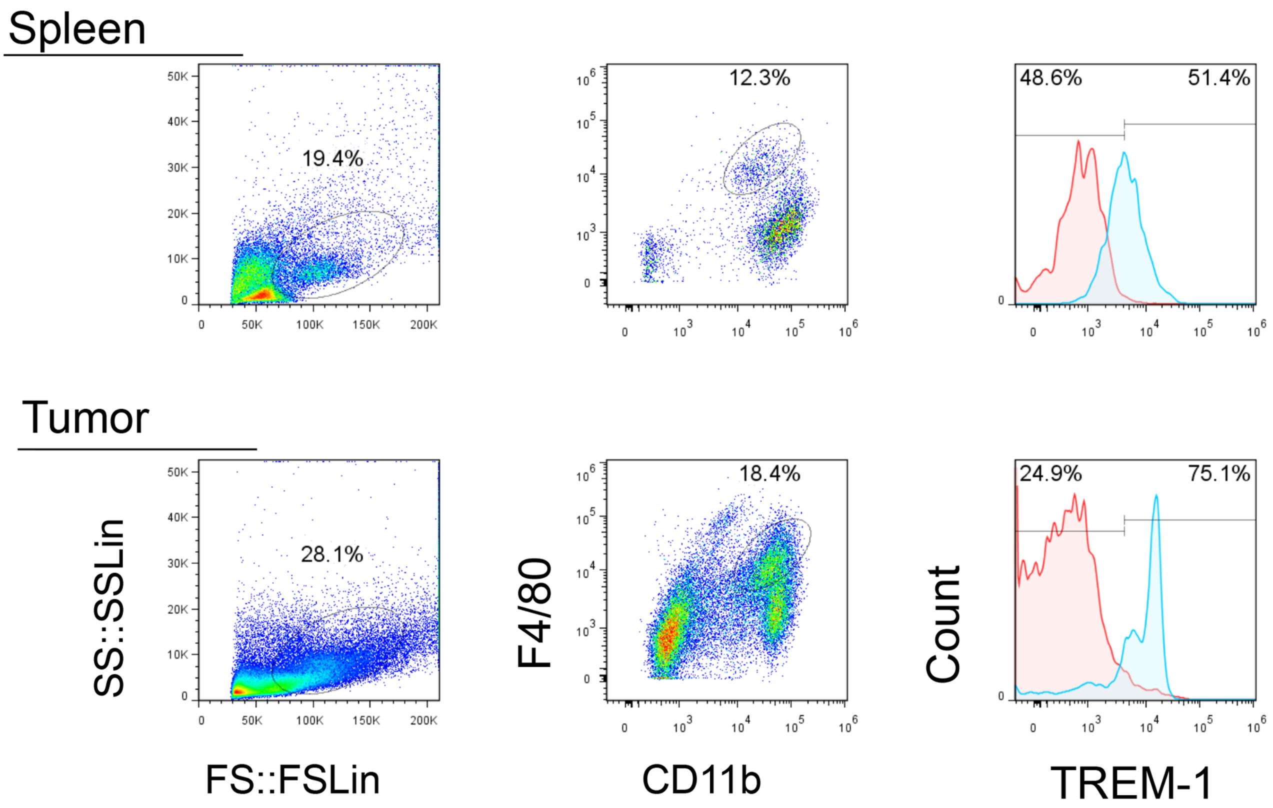

Supplementary Figure S2: Strategy for TREM-1 detection on monocytes/macrophages in mice. Flow cytometric analysis of TREM-1 expression on monocytes/macrophages of spleen or tumor tissues. Single-cell suspension was labeled with CD11b and F4/80. TREM-1 was detected (right flow diagram, right peak) and IgG was used as a control (right -flow diagram, left peak).
